# Supplementary material for: The Fusion Gene Landscape in Taiwanese Patients with Non-Small Cell Lung Cancer
Source: Cancers (Basel). 2021 Mar 16;13(6):1343. doi: 10.3390/cancers13061343 (PMC8002233; doi:10.3390/cancers13061343)
Supplement: Supplementary file 1 [file cancers-13-01343-s001.zip › cancers-1136989-supple-proofed/cancers-1136989-supple-proof/cancers-1136989-supple-proof.docx]

Supplementary Materials: The Fusion Gene Landscape in Taiwanese Patients with Non-small Cell Lung Cancer

Ya-Sian Chang, Siang-Jyun Tu, Ju-Chen Yen, Ya-Ting Lee, Hsin-Yuan Fang and
Jan-Gowth Chang


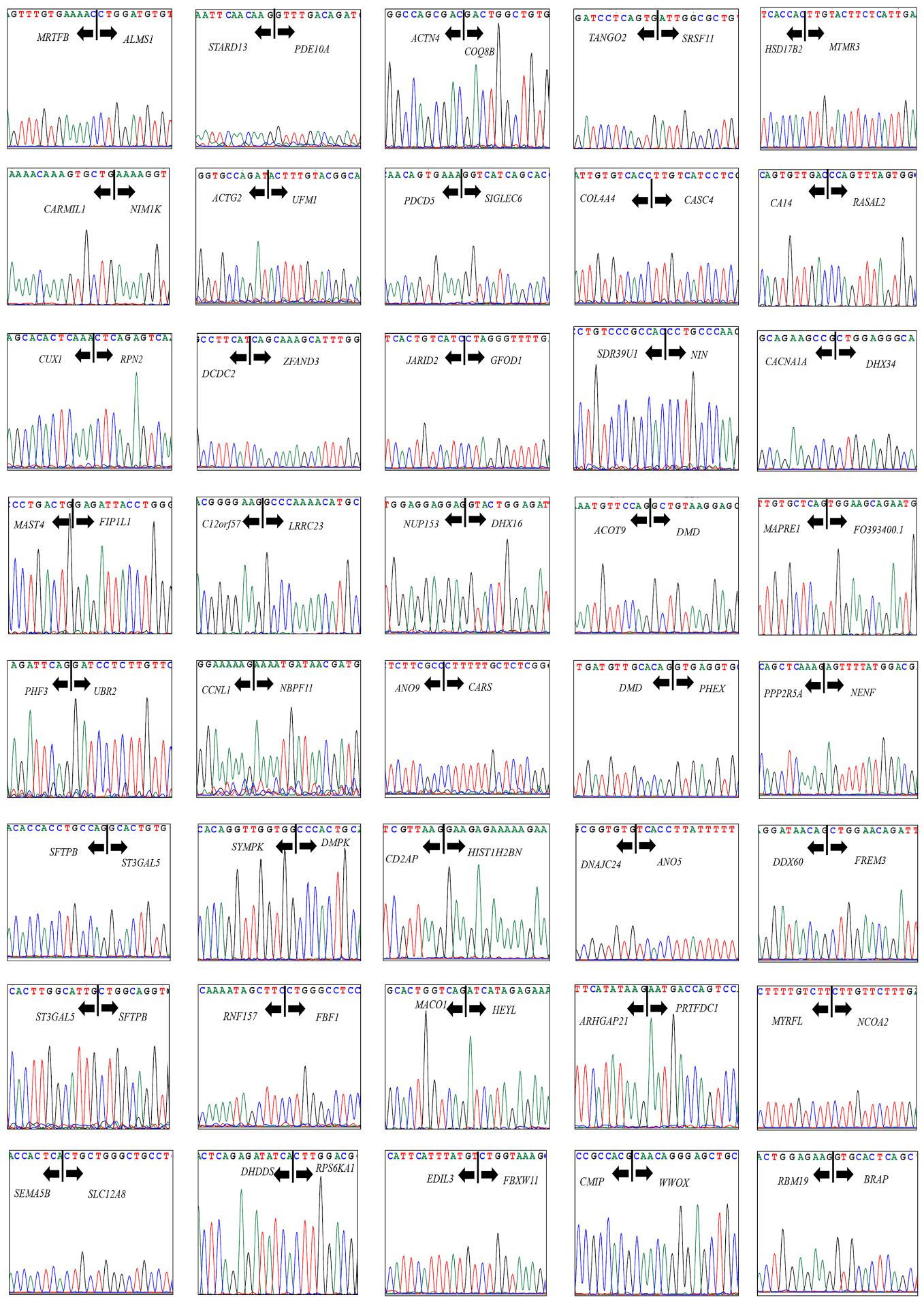


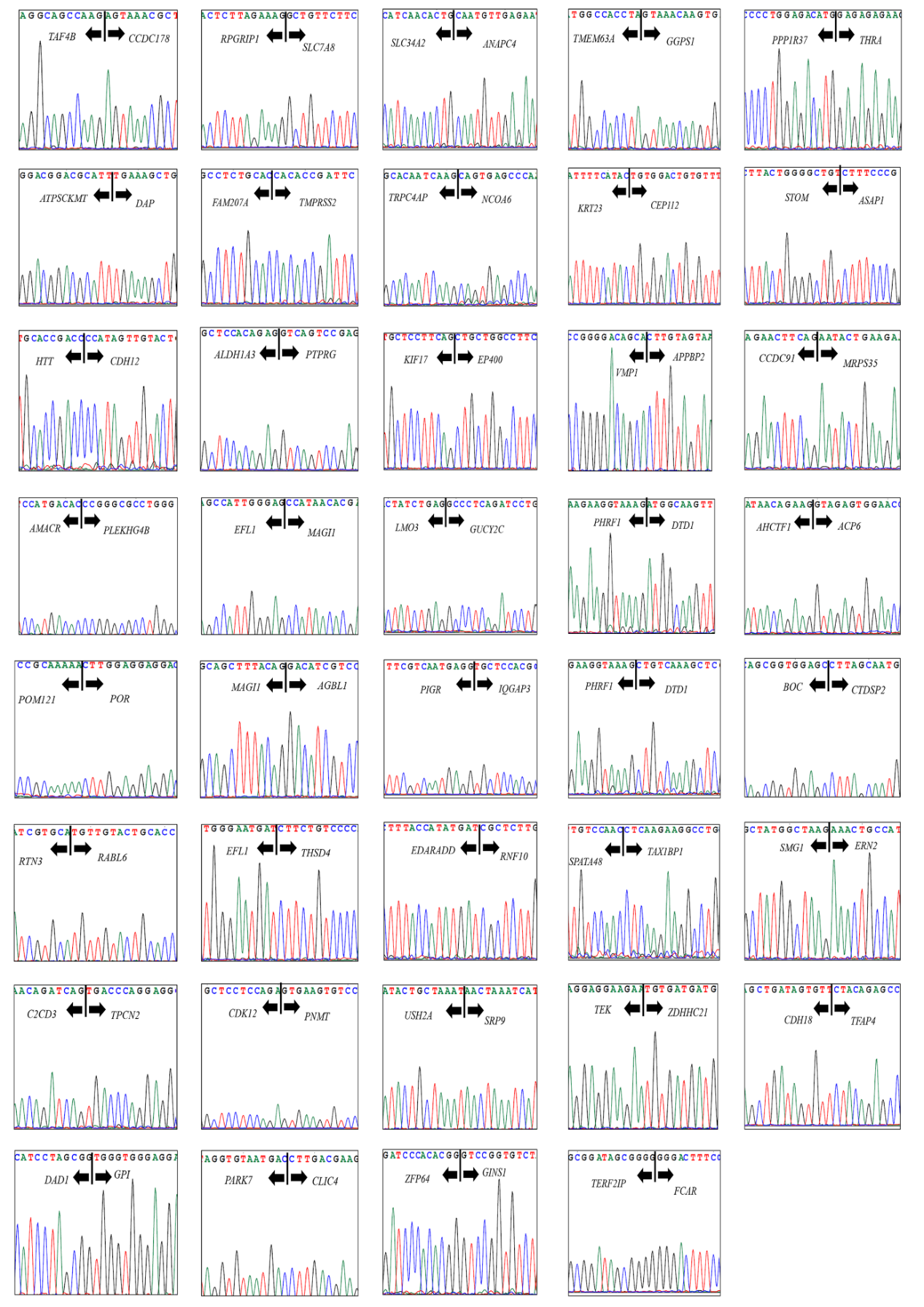


**Figure S1.** A novel fusion verified using RT-PCR and Sanger sequencing. The line represents the junction point of a fusion gene.

| 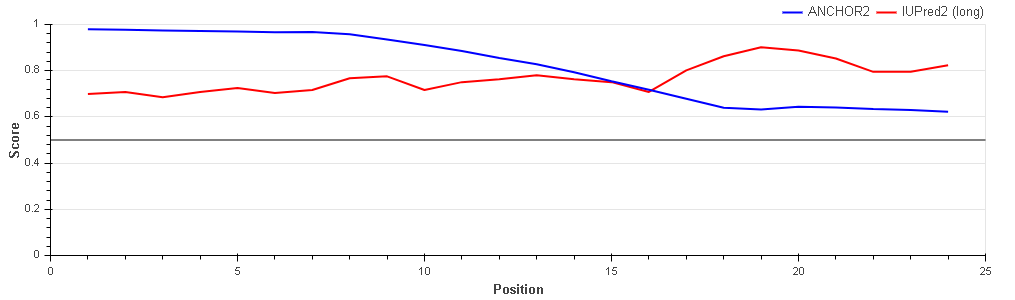 |
| --- |
| A |
| 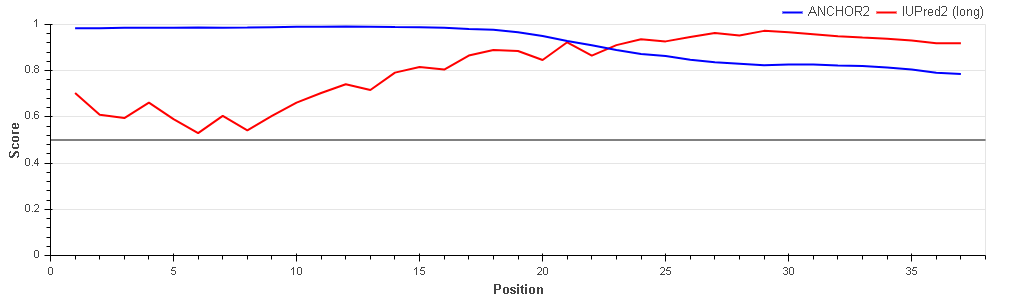 |
| B |
| 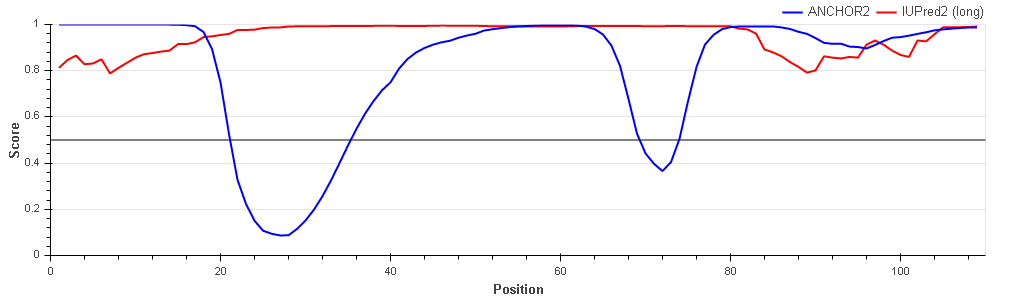 |
| C |
| 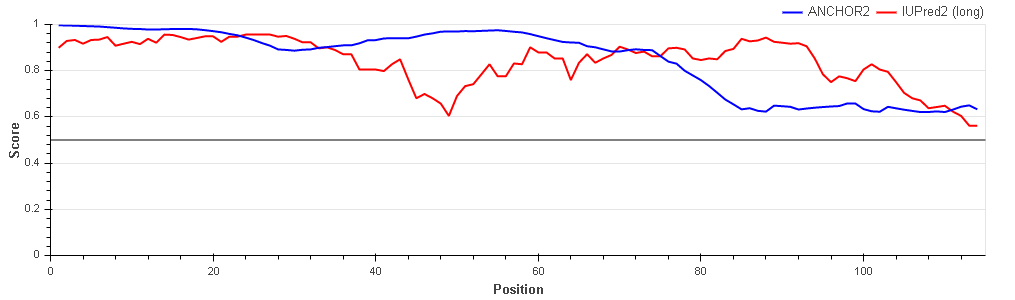 |
| D |
| 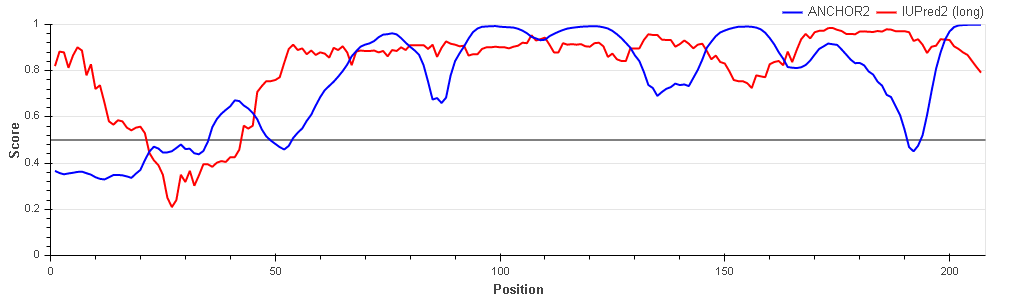 |
| E |
| 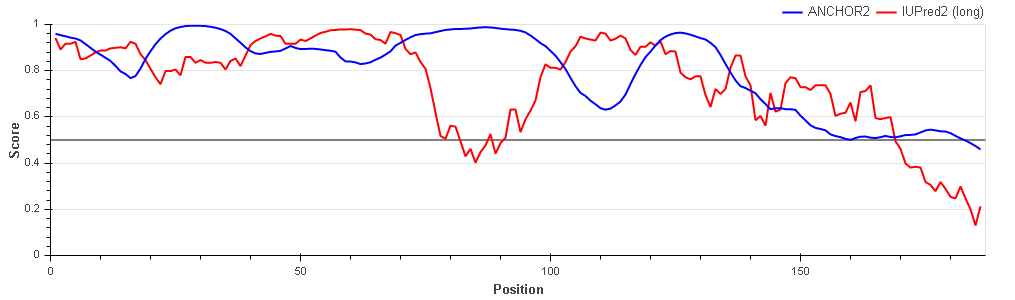 |
| F |
| 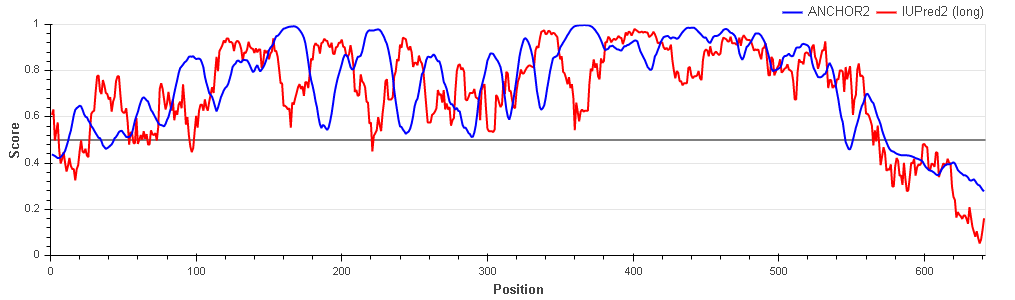 |
| G |
| 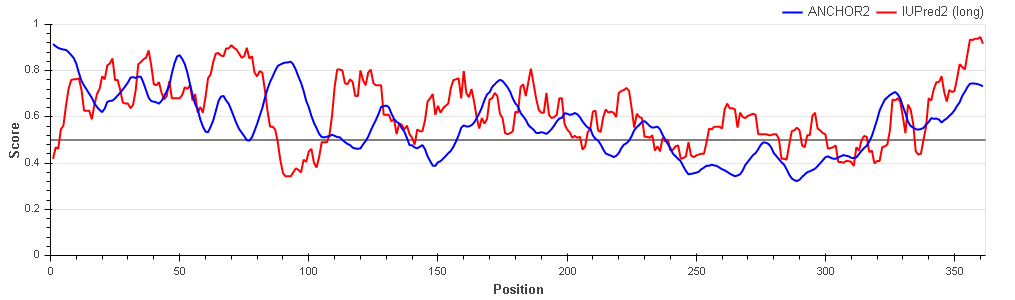 |
| H |
| 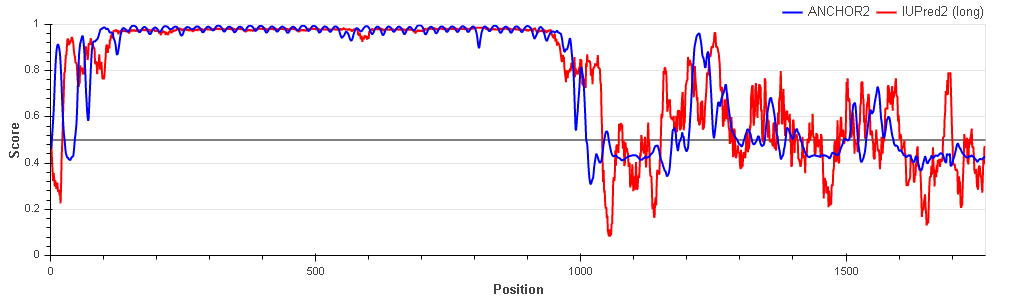 |
| I |
| 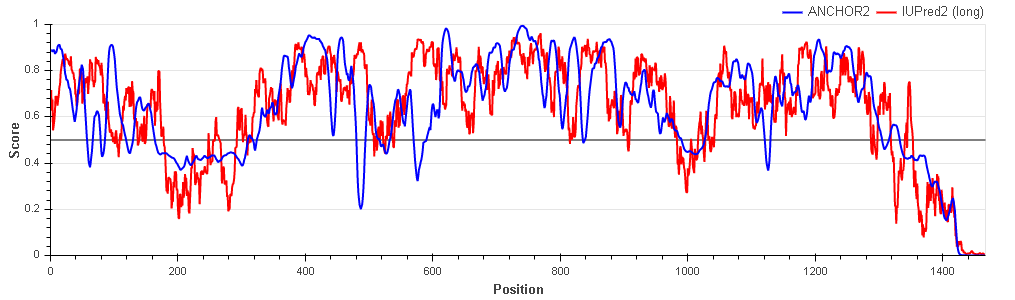 |
| J |
| 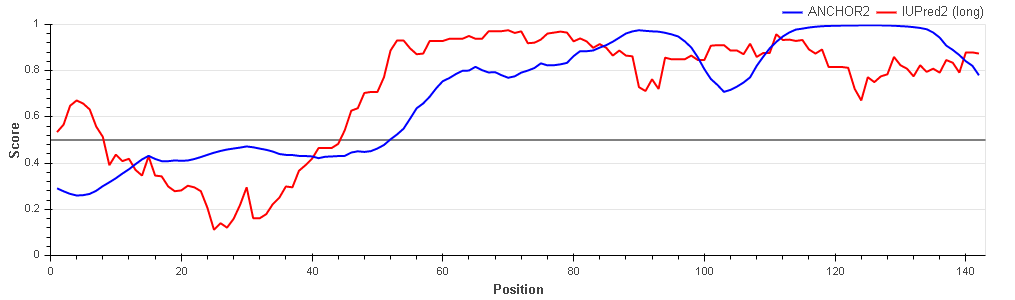 |
| K |
| 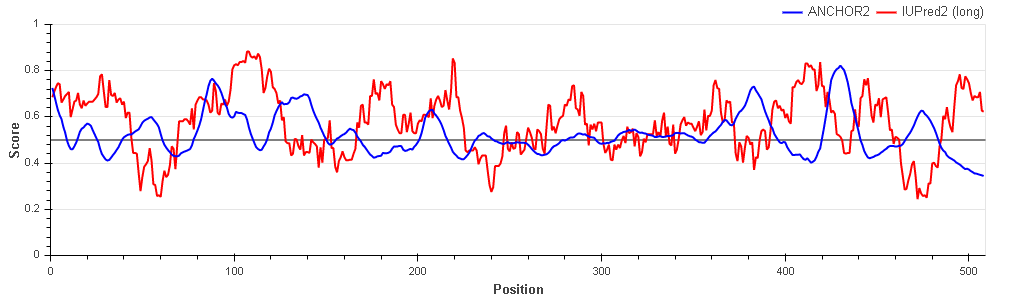 |
| L |
| 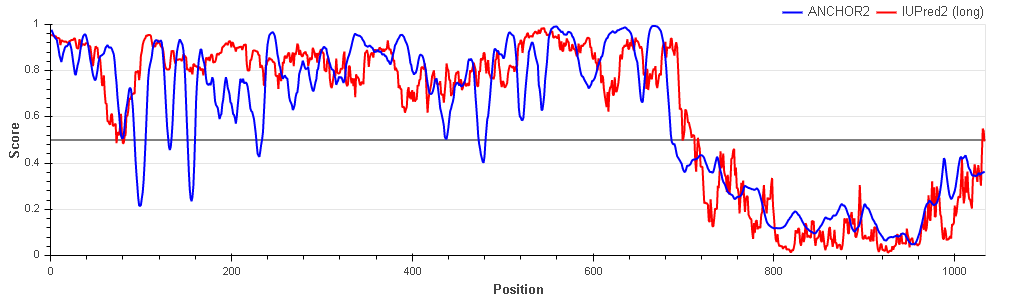 |
| M |
| 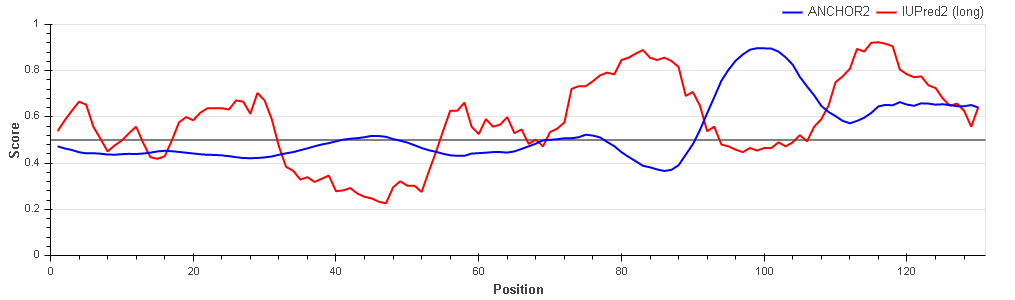 |
| N |
| 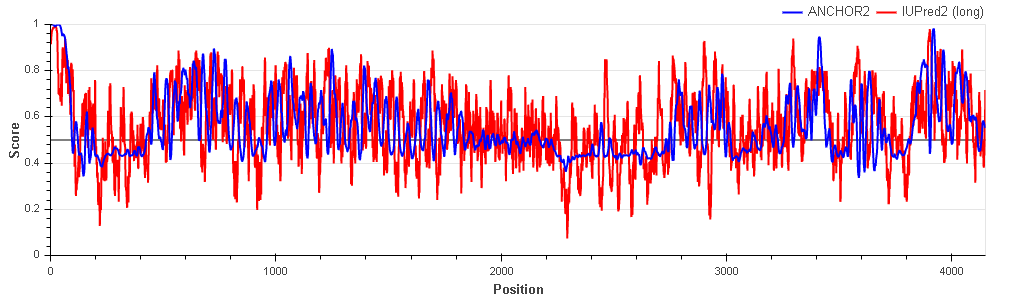 |
| O |
| 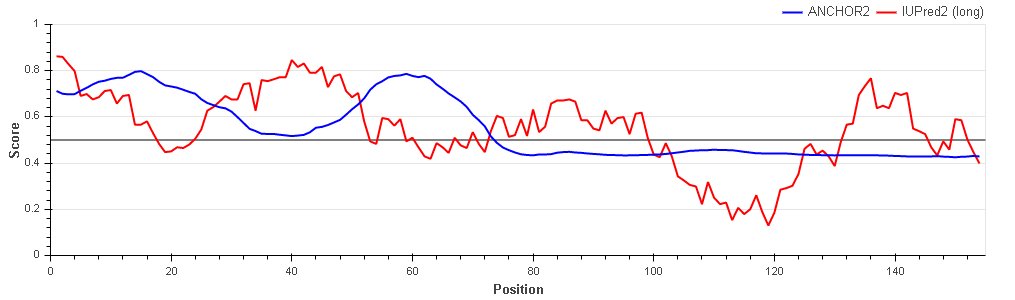 |
| P |
| 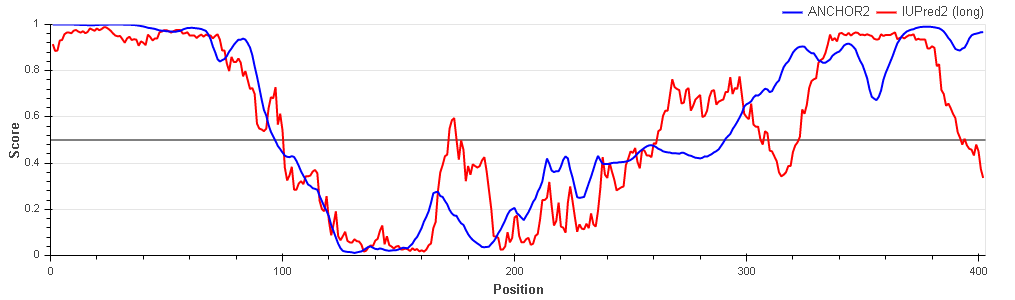 |
| Q |
| 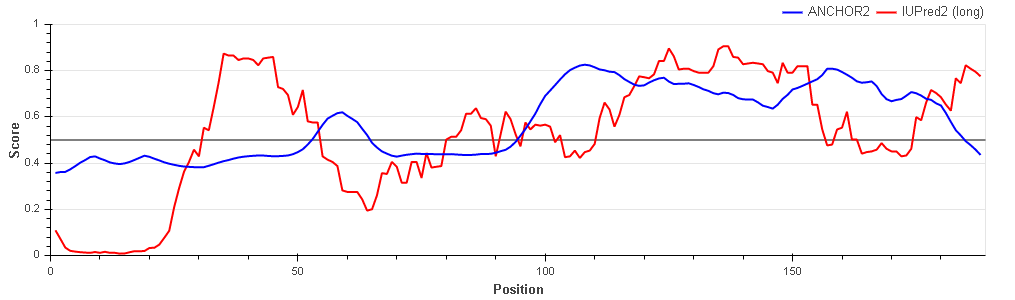 |
| R |
| 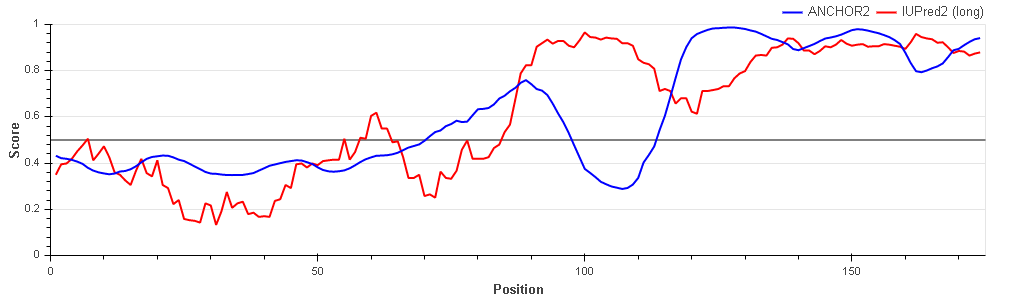 |
| S |
| 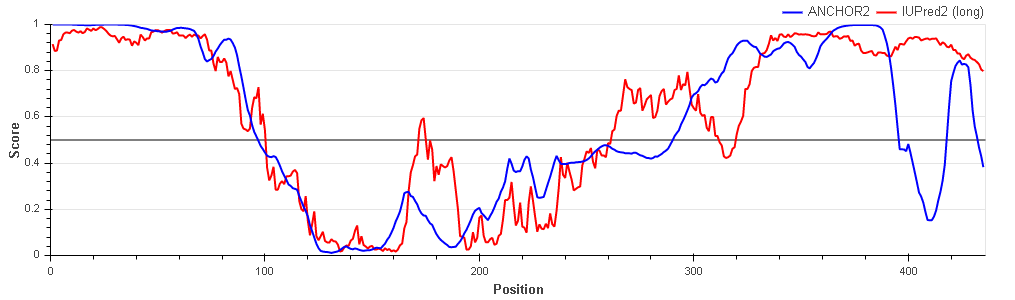 |
| T |
| 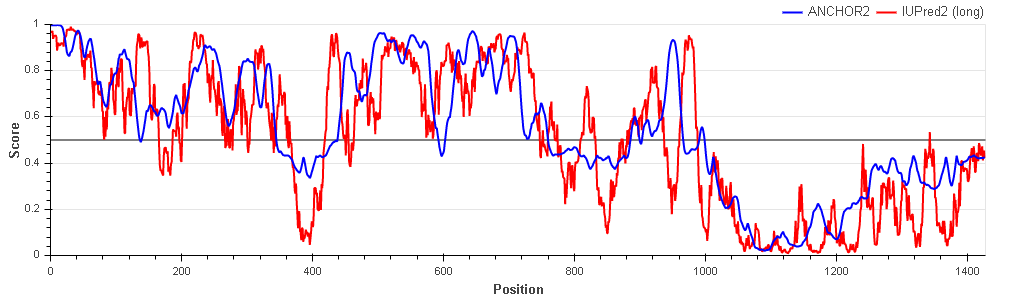 |
| U |
| 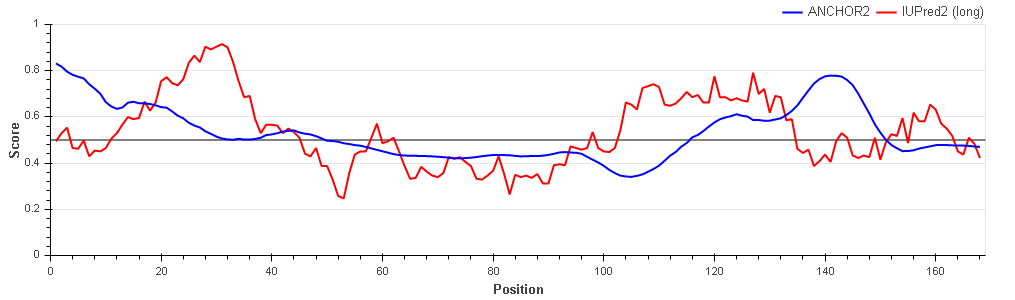 |
| V |
| 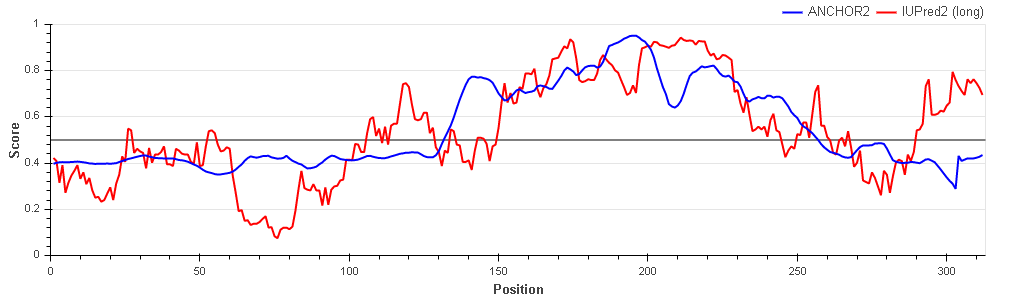 |
| W |
| 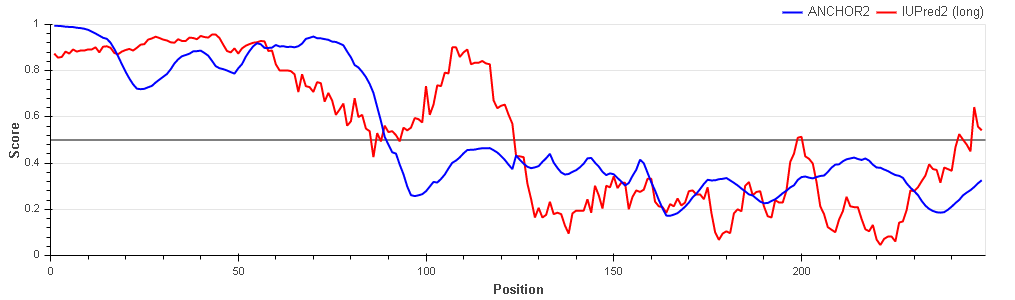 |
| X |
| 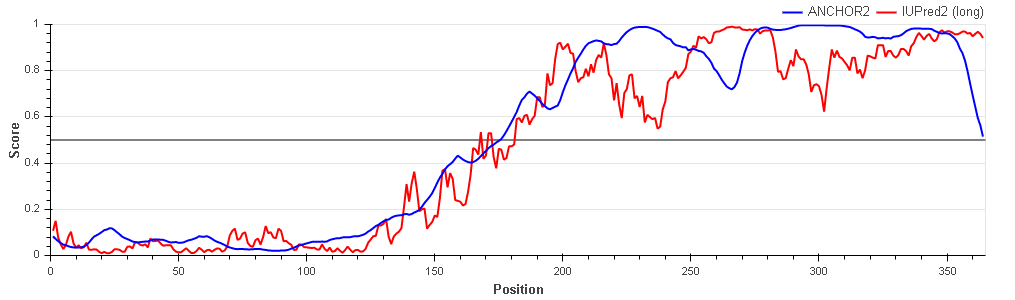 |
| Y |

**Figure S2.** Predictions of intrinsic disorder tendency of novel fusion proteins by IUPred2A (https://iupred2a.elte.hu/). Scores above 0.5 indicate disorder. (**A**) TSTD1-PFDN2 (100%, 24/24); (**B**) RPS6KA1-DHDDS (100%, 37/37); (**C**) HTT-CDH12 (100%, 109/109); (**D**) PPP1R37-THRA (100%, 114/114); (**E**) ZFAND3-DCDC2 (89.86%, 186/207); (**F**) MAST4-FIP1L1 (86.02%, 160/186); (**G**) FBF1-RNF157 (83.15%, 533/641); (**H**) RNF214-DSCAML1 (78.95%, 285/361); (**I**) MUC1-PRPF3 (78.04%, 1375/1762); (**J**) PFDN4-ASXL1 (77.97%, 1143/1466); (**K**) SFTPA1-FTL (74.65%, 106/142); (**L**) NUP153-DHX16 (70.08%, 356/508); (**M**) CDK12-PNMT (68.83%, 711/1033); (**N**) CTDSP2-BOC (65.38%, 85/103); (**O**) ALMS1-MRTFB (64.08%, 2658/4148); (**P**) PPP2R5A-NENF (62.99%, 97/154); (**Q**) PHRF1-DTD1 (62.07%, 270/435); (**R**) THSD4-EFL1 (58.51%, 110/188); (**S**) RBM19-BRAP (56.32%, 98/174); (**T**) PHRF1-DTD1 (55.22%, 222/402); (**U**) EP400-KIF17 (53.96%, 770/1427); (**V**) PDCD5-SIGLEC6 (52.38%, 88/168); (**W**) WWP2-WWOX (51.92%, 162/312); (**X**) RNF10-EDARADD (51.21%, 127/248); (**Y**) DMPK-SYMPK (51.10%, 186/364)


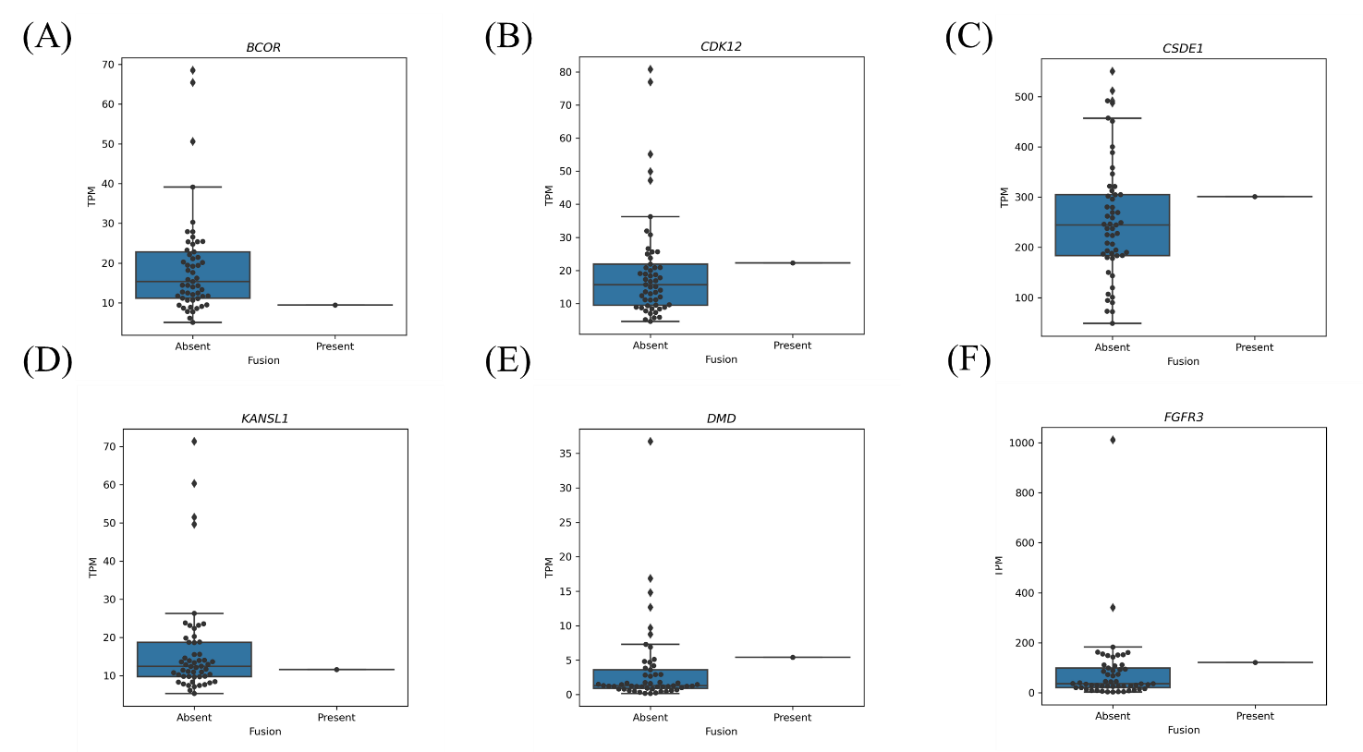


**Figure S3.** Expression levels of genes present in fusions; expression without outliers are shown. (**A**) *BCOR* expression in samples with or without *BCOR* fusion genes. (**B**) *CDK12* expression in samples with or without *CDK12* fusion genes. (**C**) *CSDE1* expression in samples with or without *CSDE1* fusion genes. (**D**) *KANSL1* expression in samples with or without *KANSL1* fusion genes. (**E**) *DMD* expression in samples with or without *DMD* fusion genes. (**F**) *FGFR3* expression in samples with or without *FGFR3* fusion genes.


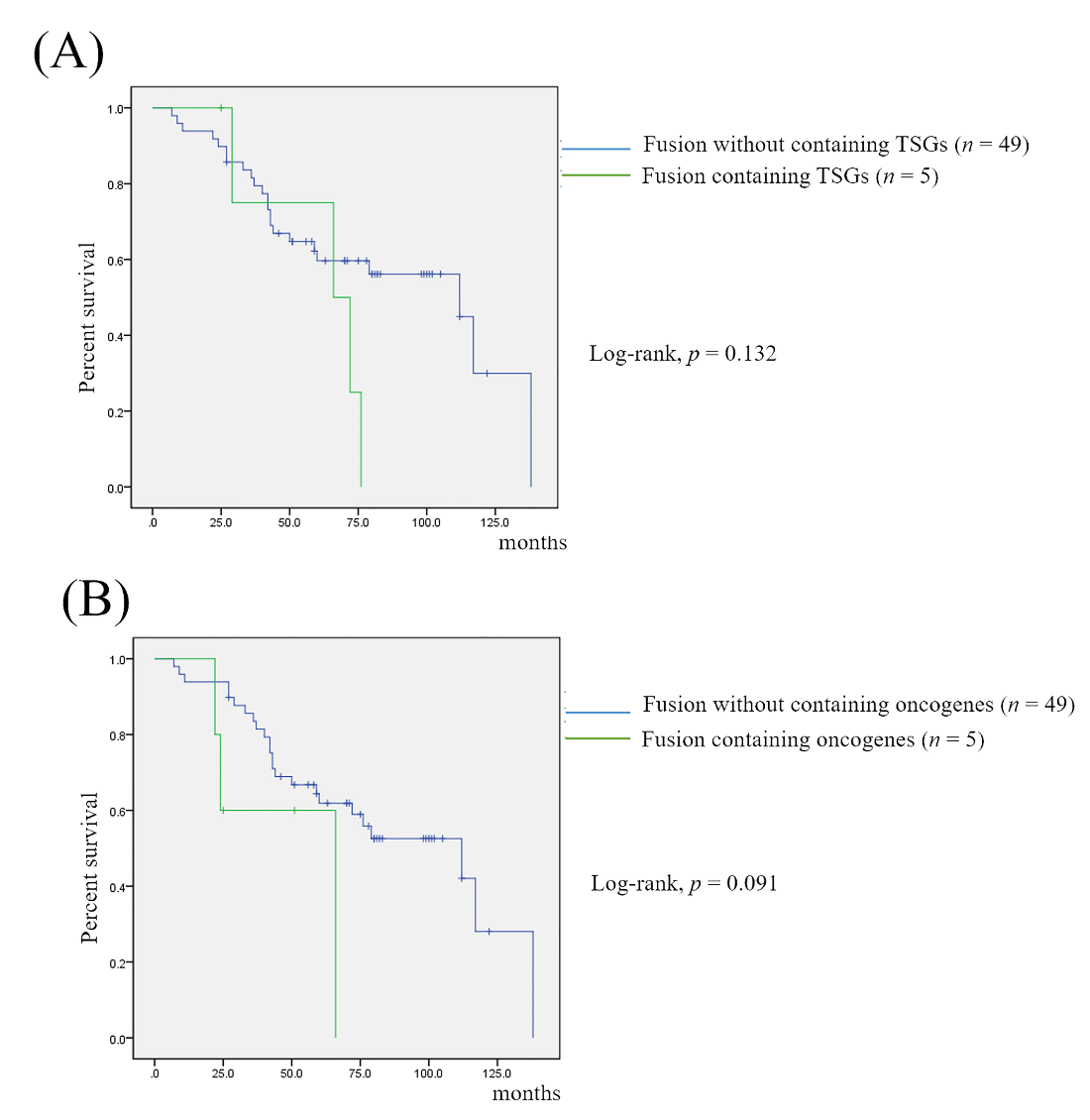


**Figure S4.** Kaplan-Meier survival curves of patients with fusions containing (**A**) TSGs (**B**) oncogenes.


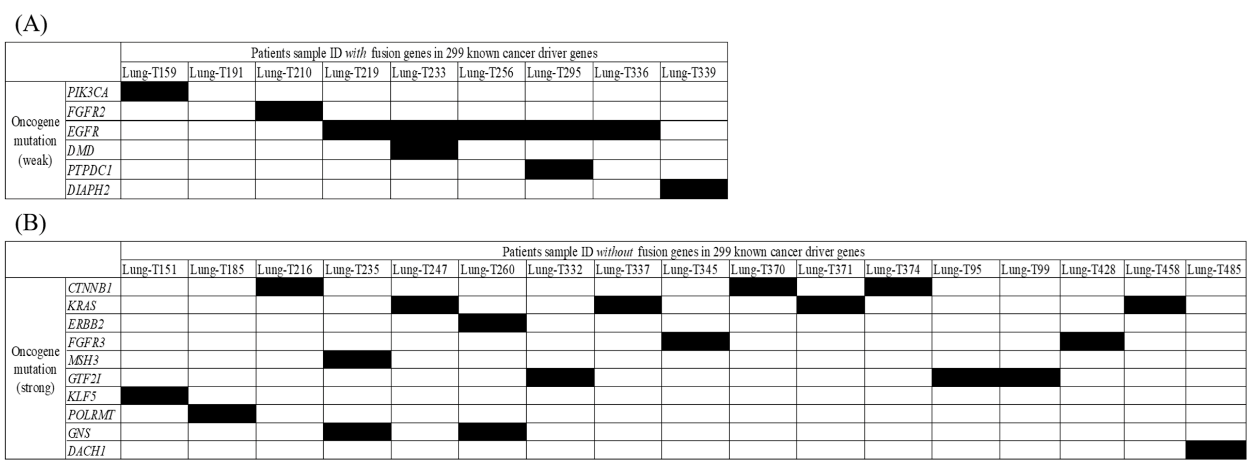


**Figure S5.** Schematic overview of oncogene mutation profiling. (**A**) The numbers on top designate each patient sample ID with oncogene mutations and fusion genes; Lung-T159, *FGFR3*-*TACC3*; Lung-T210, *PFDN4*-*ASXL1*; Lung-T219, *CSDE1*-*TERT*; Lung-T233, *DMD*-*PHEX*, *ACOT9*-*DMD* and *BCOR*-*DMD*; Lung-T256, *DHX34*-*CACNA1A* and *KANSL1*-*AC091132.4*; Lung-T295, *NOTCH2*-*SHE*; Lung-T336, *NOTCH2*-*LINC00623*; Lung-T339, *KIF5B*-*RET* (**B**) The numbers on top designate each patient sample ID with oncogene mutations but without fusion genes.

| 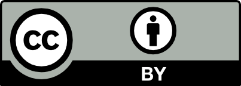 | © 2021 by the authors. Licensee MDPI, Basel, Switzerland. This article is an open access article distributed under the terms and conditions of the Creative Commons Attribution (CC BY) license (http://creativecommons.org/licenses/by/4.0/). |
| --- | --- |
